# A Tablet App Supporting Self-Management for People With Dementia: Explorative Study of Adoption and Use Patterns

**DOI:** 10.2196/14694

**Published:** 2020-01-17

**Authors:** Laila Øksnebjerg, Bob Woods, Kathrine Ruth, Annette Lauridsen, Susanne Kristiansen, Helle Dalsgaard Holst, Gunhild Waldemar

**Affiliations:** 1 Danish Dementia Research Centre Department of Neurology, The Neuroscience Centre Rigshospitalet Copenhagen Denmark; 2 Dementia Services Development Centre Wales Bangor University Bangor United Kingdom; 3 Department of Neurology Zealand University Hospital Roskilde Denmark; 4 Dementia Clinic, Department of Neurology Aalborg University Hospital Aalborg Denmark; 5 Faculty of Health and Medical Sciences University of Copenhagen Copenhagen Denmark

**Keywords:** dementia, technology, information technology, self-help devices, app, self-management, rehabilitation, memory, caregivers

## Abstract

**Background:**

Assistive technology (AT) is rapidly emerging within dementia care and support. One area of AT application is support of people with dementia in compensating for cognitive symptoms and thereby promoting their self-management. There is, however, little evidence for the applicability, usability, and effectiveness of AT for people with dementia, and there is a need to identify factors that can promote adoption.

**Objective:**

This study aimed to (1) evaluate the applicability and usability of an app, tailor-made for people with dementia; (2) explore factors affecting adoption; (3) explore the possible influence of caregiver involvement; and (4) contribute to process evaluation of the intervention.

**Methods:**

The ReACT (*Rehabilitation in Alzheimer's disease using Cognitive support Technology*) app was designed as a holistic solution to support memory and structure in daily living. Persons with dementia had access to a personal user account, and family caregivers were given a parallel login. Written and Web-based materials were provided to support self-applied implementation. A mixed methods design was applied to explore adoption and use patterns, including background and disease-related data, qualitative data from a survey, and log data. Adoption was defined as the use of the app over a period of ≥90 days.

**Results:**

Data from 112 participants and 98 caregivers were included. Shorter time from diagnosis (U=595; *P*=.046; *r*=0.19) and caregiver activating the app (*P*=.02) had a significant impact on the participant adoption status. Logistic regression analysis showed that if caregivers had activated the app, the participant was five times more likely to become an adopter (odds ratio 5.1, 95% CI 1.29-19.99; *P*=.02). However, the overall predictive power was low, and there was a wide variation in background and disease-related characteristics among adopters. The level of experience and skills in tablet use were not significantly different between adopters and nonadopters. Adopters generally rated the app high on usefulness, satisfaction, and ease of use (rated on the USEdem questionnaire). Their scores were significantly higher compared with nonadopters (U=5.5; *P*=.02; *r*=0.64). Analysis of use patterns showed that all functionalities of the app were used among adopters.

**Conclusions:**

For participants who became adopters, the ReACT app and the methods for self-applied implementation were applicable. However, the results were also in accordance with the well-known challenges of nonadoption and nonadherence to digital health interventions. The study provided insight into the importance of timely introduction and caregiver support for adoption of AT among people with dementia. It also underlined the high complexity of personal and contextual factors that influence adoption. These complex factors need to be considered when designing and implementing AT for people with dementia.

## Introduction

Globally, the number of people living with dementia is increasing rapidly [1], and it is a global concern that in the near future, we may not have enough resources to meet their need for support and care [2]. These challenges call for efficient and flexible solutions, and the progressive digitalization and use of technology are seen as promising solutions [2,3]. In the last decade, there has been a steadily growing emphasis on assistive technology (AT) in dementia care and support, both from the industry, governments, and organizations and in research [4-6]. It is assumed that AT has great potential to support cognition and can be used to compensate for cognitive decline, which is a core symptom across dementia diseases, and thereby promote self-management of people living with dementia [7-9].

AT comprises a variety of solutions, ranging from basic everyday low-technology devices to advanced technology devices [10]. In the field of dementia, AT based on information and communication technology (ICT), such as apps applied on touchscreen devices, has expanded [6]. The focus of this study is ICT-based AT, and for the remainder of the paper, this will be the kind of AT referred to.

The optimism on the potential of AT to support people with dementia is unfortunately not based on strong evidence. There is a great need for research addressing applicability, usability, and effectiveness of AT for people with dementia [8,11,12], and when designing and conducting research within this field, a complex set of preconditions must be considered. First, this field of research is subject to the same challenges as other digital health interventions. The relatively high rate of nonadoption and nonadherence, referred to as *the law of attrition* [13], is a natural and typical feature of digital health interventions in all medical fields [14,15]. Consequently, the pattern of dropout rates in research addressing these kinds of interventions is quite distinct from other types of research, such as drug trials or studies of psychosocial interventions. This requires a different approach to research methodology, for example, by including detailed log data analysis to explore user patterns and analyze characteristics of individuals who successfully adopt and adhere to the technology [13]. Such perspectives and methodologies are often not included in research addressing AT for people with dementia, where more traditional quantitative and qualitative outcome measures have often been applied to assess usability and impact [8]. Another challenge is the progressive cognitive symptoms of dementia diseases that reduce the ability of people with dementia to adopt and adhere to AT. Therefore, it is essential to explore factors (for instance, caregiver support and tailor-made interventions) that can support and promote their adoption and adherence. Consequently, to explore the potential of AT for people with dementia and to provide evidence for specific solutions, study designs have to incorporate these complex preconditions. An essential step is the use of mixed methods when assessing applicability, usability, and effectiveness, including log data analysis.

This paper has presented data from the third substudy of the research project, *Rehabilitation in Alzheimer's disease using Cognitive support Technology* (ReACT). In the first substudy, the ReACT app was created through an iterative user-centered design process [16]. The app was tailor-made to support self-management of people with dementia, mainly by supporting various aspects of prospective and retrospective memory and structuring of daily activities. People with early stage Alzheimer disease were considered the main group of end users during the design phase, and the applicability and usability of the app for people with Alzheimer disease have been investigated in a second substudy [17]. This third substudy was conducted to investigate the applicability and usability of the app in a large mixed cohort of people with dementia, representing various etiologies and backgrounds. To the best of the authors’ knowledge, this paper is the first to present detailed data, including an analysis of log data, from a study where AT tailor-made for people with dementia has been disseminated to a large heterogeneous group of people with dementia.

Accordingly, the aims of the study were to (1) investigate the applicability and usability of the ReACT app to a mixed population of people with dementia, (2) investigate user patterns and factors influencing adoption and nonadoption, (3) explore the possible influence of caregiver involvement on participants’ adoption of the app, and (4) contribute to process evaluation of the app and methods used for deployment and adoption to guide future adaption of the app and methods of implementation.

## Methods

### Participants

Participants were recruited from 9 Danish memory clinics. The aim was to recruit a broad variety of people with dementia, and therefore, few inclusion criteria were specified. Participants were eligible if they (1) were patients in the memory clinics, (2) were motivated to try using the app, and (3) had access to a tablet where the app could be installed (iPad). It was not mandatory to have a caregiver as coparticipant, but if they were accompanied by a family caregiver when visiting the clinic, the caregiver was invited as coparticipant. There were no inclusion criteria related to age, language, or other personal or disease-related characteristics, and participants were not required to have had a final diagnosis at the time of inclusion. As the intervention addressed people with dementia as primary participants, they are referred to as participants in this paper, and the coparticipating caregivers are referred to as caregivers.

Information about the study was presented to patients in the memory clinics. Posters and flyers presenting the app and the study were available in waiting areas and introduced by staff. Eligible participants who showed interest in the study were given a detailed oral introduction and additional written material describing the details of the study. Participants and caregivers were then given an opportunity to deliberate before deciding whether to participate in the study.

Participants were recruited from June 2017 to February 2018, and during this period, 116 participants and 98 caregivers were enrolled.

The regional scientific ethical committees of the Capital Region of Denmark (protocol number H-15005558) evaluated the study protocol and decided that the study did not need approval because it was not considered to be within the framework of biomedical research. All participants received oral and written information about the study objectives and methods, and all participants gave written informed consent.

### Intervention

**Figure 1:**
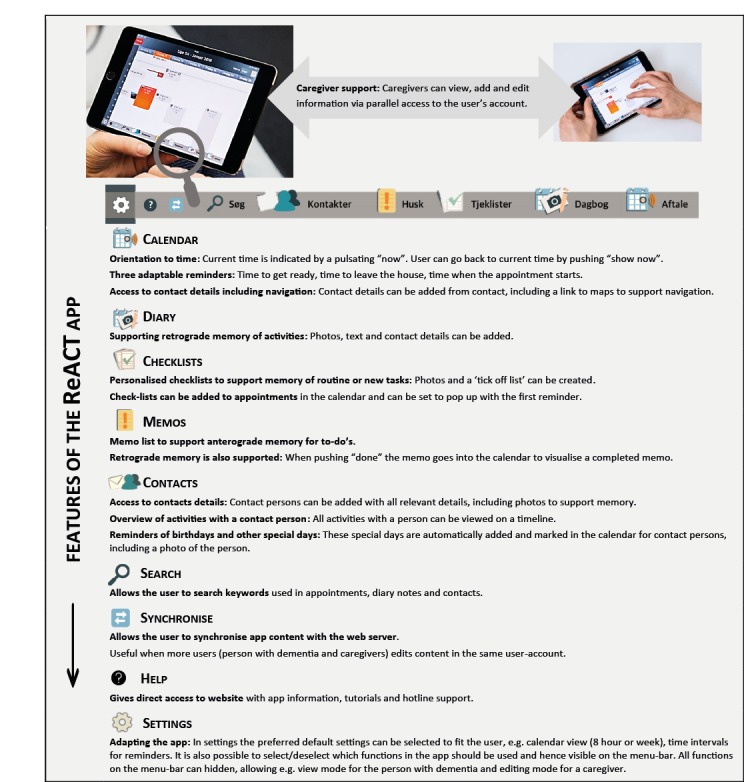
Features of the ReACT app.

Participants and caregivers were given access to the ReACT app after being included in the study. The specific features of the ReACT app are illustrated and specified in Figure 1. As described, the app was a holistic solution, comprising a calendar that interacts with other features (eg, diary notes, contacts, checklists, and memos). It was designed as a cloud-based native app for a tablet computer and could be accessed from an iPad.

As described in Figure 1, the participants had access to a personal user account, and caregivers could support the use of the app via a parallel login to this account, allowing them to add and edit content. Usernames and access codes were provided separately to participants and caregivers to enable monitoring of log data from both. For data protection, these codes were provided in sealed envelopes to the participant and caregiver, and staff at the memory clinics had no access to this information. In case of need for backup information on usernames and access codes, these could only be provided if a participant or caregiver contacted the principal investigator of the study directly.

Participants and caregivers were provided written material to support their self-applied implementation of the app. This written material had been validated as applicable for a person with early stage dementia during the second substudy of the ReACT research project [17], where people with early stage Alzheimer disease and caregivers were consulted when developing these materials. Two leaflets were provided: one leaflet gave instructions on how to download and activate the ReACT app on a tablet, whereas another leaflet gave a brief introduction to the functionalities of the app and gave advice on how the app could be used in various everyday situations, including how caregivers could support the use of the app. Details on telephone and email hotline support were also included. Hotline support was accessible within working hours throughout the study period.

A help feature was also built into the app, as illustrated in Figure 1. When tapping this icon on the app’s menu bar, the user was directed to a website with information, video tutorials, and hotline details.

### Assessments

#### Baseline Characteristics

Demographic information for both participants and caregivers was collected at the time of inclusion. Data included participant’s age, gender, and education; caregiver’s gender; and the relation between participant and caregiver. In addition, data from participants’ medical records were included to document diagnosis, time of diagnosis, and the most recent score on the Mini-Mental State Examination (MMSE) [18], a brief assessment of cognitive function.

#### Log Data

App usage was monitored through data logs from each participant’s and caregiver’s use of the app. The log files provided data on participant’s and caregiver’s actions in the app and provided information on action types and timestamps for these actions. The action types that were logged were activating the app; using adaptive features; and activities related to appointment, diary note, memos, checklist, and search features.

Adoption of the app was defined as a minimum period of 90 days between the first and last use of the app. This criterion was set based on results from the previous substudy [17], showing that a person with early stage dementia could adopt the app and use it independently after a period of 90 days of introduction and familiarizing with it.

#### Survey

A Web-based survey was conducted to collect additional background information and to collect feedback on the app. It was distributed via email 3 to 4 months after inclusion in the study. In cases where email correspondence was unsuccessful, a printed version of the survey was sent out by mail.

Two versions of the survey were distributed: one for participants and another by-proxy version for caregivers. It was constructed in an adaptable manner, enabling specific questions being directed at those who had used the app and those who had not. The survey included questions with a fixed set of possible answers. For nonusers, the questions addressed the level of skills to use a tablet and reasons for not using the app. For users, the questions addressed methods used when learning how to use the app and the level of skills to use a tablet. Moreover, 2 optional text boxes were also included, allowing additional comments on reasons for not using the app and general feedback on the app. The general feedback mainly addressed specific technical and functional issues of the app, which is not within the scope of this paper.

In those cases where the participant had tried using the app, the USEdem questionnaire [17] was included in the survey, and a by-proxy version was delivered to caregivers. The USEdem questionnaire is a modified version of the Usefulness, Satisfaction, and Ease of Use Questionnaire [19], which was designed to assess usefulness, satisfaction, and ease of use of technology. The USEdem questionnaire was designed and applied in a previous substudy of the ReACT research project [17]. This modified version contains 12 items and was adapted to be applied to people with dementia [17]. Scores on each item range from 1 to 5 on a Likert scale, with a total score between 12 and 60, higher scores indicating higher ratings.

#### Data Analysis

Quantitative data were analyzed using IBM SPSS Statistics version 22. Baseline characteristics and log data from adopters were explored with descriptive statistics. Possible between-group differences on baseline characteristics, log data, and data from the surveys were analyzed using nonparametric chi-square tests for categorical variables, and for continuous variables, Kruskal-Wallis tests, Mann-Whitney U tests, or Fisher exact tests were used as appropriate. Logistic regression was conducted to explore whether baseline characteristics predicted adoption status.

Tests of significance were performed 2-tailed, with a significance level of .05. Imputed values for missing data on background characteristics were calculated following standard procedures for multiple regression modeling.

Qualitative data from surveys, from the textbox allowing feedback on reasons for not using the app, were processed and summarized in themes, as outlined in constant comparison analysis [20].

## Results

### Principal Results

Data from 112 participants and 98 caregivers were included in data analysis. No participants or caregivers withdrew their consent to participate in the study, but because of insufficient background information from 4 participants, these were excluded from the original sample of 116 participants.

Log data monitoring the use of the app were collected for all participants and caregivers for a maximum of 90 days; hence, the intervention period for this study was 90 consecutive days after activating the app or 90 days from the inclusion of those who did not activate the app. Additional follow-up data are not within the scope of this paper.

Data from the surveys were obtained from 35 participants and 30 caregivers, and 19 of these cases were overlapping, with a reply from both. Of 35 participants, 14 had support from a caregiver when answering the survey, and in 2 cases, a caregiver had answered the participant survey on behalf of the participant; these 2 were excluded, leaving 33 participant and 30 caregiver replies for data analysis. Included in this were data on the USEdem questionnaire from 14 participants and 9 caregivers from cases where the participant had tried using the app were included.

### Participants’ Characteristics

The characteristics of participants and caregivers are summarized in Table 1. These data show that participants were quite heterogeneous with regard to age, education level, months since diagnosis, and most recent MMSE score. Most participants had a diagnosis of Alzheimer disease. People with diagnoses of vascular dementia and other less common dementia, such as frontotemporal dementia, were also represented. Those with a diagnosis of mild cognitive impairment were also a part of the participants. A fairly large group of participants were categorized as other and included, for example, participants with an unspecified dementia diagnosis or cognitive symptoms caused by stroke. Gender was almost equally represented, but with a slightly greater proportion of men among participants. A dyad of participants and caregivers were included in 87.5% (98/112) of the cases, and most of these caregivers were spouses.

**Table 1:** Characteristics of participants and caregivers and differences between adopters and nonadopters.

Characteristics		All participants (N=112)	Adopters^a^ (N=18)	Nonadopters^a^ (N=94)		*P* value
Age (years), mean (SD; range)		68 (8.8; 39-86)	69 (10; 52-82)	68 (8.6; 39-86)		.86
**Gender, n (%)**						
	Women	49 (43)	9 (50)	40 (43)		.51
**Years of education, n (%)**					.36	
	≤10	16 (14)	3 (17)	13 (14)		
	11-12	23 (20)	3 (17)	20 (21)		
	13-14	25 (22)	2 (11)	23 (25)		
	15-16	29 (25)	8 (44)	21 (22)		
	≥17	19 (16)	2 (11)	17 (18)		
**Diagnosis, n (%)**					.29	
	Alzheimer disease	65 (58)	12 (67)	53 (56)		
	Vascular dementia	2 (1)	0	2 (2)		
	Dementia with Lewy bodies	1 (0)	1 (6)	0		
	Frontotemporal dementia	3 (2)	0	3 (3)		
	Mild cognitive impairment	9 (8)	2 (11)	7 (8)		
	Other^b^	27 (24)	2 (11)	25 (26)		
	Unresolved^c^	5 (4)	1 (6)	4 (4)		
Months since diagnosis, mean (SD; range)		12 (15; 0-73)	6 (7; 0-25)	16 (14; 0-73)		.046
Mini-Mental State Examination score^d^, mean (SD; range)		25 (4.2; 11-30)	25 (5; 11-30)	25 (4; 11-30)		.69
**Caregiver included, n (%)**						
	Yes	98 (87)	15 (83)	83 (88)		.70
**Caregiver gender, n (%)**						
	Woman	58 (59)	8 (44)	50 (60)		.78
**Caregiver relation, n (%)**					.30	
	Spouse	81 (83)	11 (73)	70 (84)		
	Son or daughter	13 (13)	4 (27)	9 (11)		
	Other	4 (4)	0	4 (5)		
**Caregiver adopter or nonadopter of the app^a^, n (%)**						
	Adopter	7 (6)	3 (17)	4 (4)		.08
**Caregiver activated the app^e^, n (%)**						
	Yes	21 (19)	8 (44)	13 (14)		.02

^a^Adoption was defined as the use of the app for ≥90 days.

^b^For example, stroke, Huntington disease, and unspecified dementia diagnosis.

^c^Participants who were not diagnosed at the time of inclusion.

^d^Higher scores indicate higher attainment. The scores on Mini-Mental State Examination (MMSE) are the most recent score documented in the participants’ medical record. Data on months between the latest MMSE score and study inclusion showed MMSE were, on average, conducted 9 months before inclusion (SD 10; range 0-47), and 89 (89/112, 79.4%) of the MMSEs were conducted less than 12 months before inclusion.

^e^Includes all levels of caregiver adoption status.

### Adoption

The details of adoption status and period of adherence are specified in Table 2, showing that 18 (16%) participants and 7 (7%) of the caregivers became adopters. Overall, 47 (42%) participants and 78 (80%) caregivers never activated the app. However, if adoption status was based only on those who had activated the app, 28% of participants and 35% of caregivers were adopters. The aim of this study was to explore nonuse, abandonment, and adoption of the app, and therefore, data from the entire population were included in data analysis when applicable.

Caregivers had also activated the app in 44% of the 18 cases where the participants became adopters, and in 3 of these cases, both the participant and caregiver were adopters. In 4 additional cases, the caregiver became adopter without the participant becoming adopter.

As summarized in Table 1, when comparing adopters with nonadopters, results showed that time from diagnosis was significantly lower for adopters (median 4 months) than for nonadopters (median 8 months; U=595; *P*=.046; *r*=0.19). There was also a significant association between participants’ adoption status and whether caregivers had activated the app (*P*=.02; FET). There were no significant associations for other characteristics.

An exploratory logistic regression analysis was performed to assess the impact of baseline characteristics and caregiver’s app activities on participant adopter status. As shown in Table 3, caregivers having used the app was a significant predictor of a participant’s adoption status (odds ratio 5.1, 95% CI 1.29-19.99; *P*=.02). Results indicated that a participant was 5 times more likely to become an adopter when a caregiver had engaged in activating the app.

Data from the survey provided additional information on adopters and nonadopters. As summarized in Table 4, there were no significant differences in either disease-related or background characteristics between participants who replied to the survey and those who did not reply. However, months since diagnosis was close to significance (*P*=.06).

The survey gave opportunity to further explore reasons why participants did not use the app or become an adopter. As outlined in Table 5, there were no significant differences between adopters and nonadopters regarding their level of experience, skills, and need for help when using a tablet, both when rated by the participants and caregivers.

**Table 2:** Adoption status and period of adherence among participants and caregivers.

Participants and caregivers		Participants (N=112)	Caregivers (N=98)
**Adoption status, n (%)**			
	Never used the app (0 days)	47 (42)	78 (80)
**Activated the app, n (%)**			
	Short use (1-10 days)	19 (17)	7 (7)
	Early abandonment (11-31 days)	11 (10)	2 (2)
	Late abandonment (32-89 days)	17 (15)	4 (4)
	Adopter (≥90 days)	18 (16)	7 (7)

**Table 3:** Logistic regression: Impact of baseline characteristics and caregiver’s app activities on participant adoption status.

Included	Beta (SE)	*P* value	Odds ratio (95% CI)
Participant’s age	−.03 (0.03)	.29	0.97 (0.91-1.03)
Participant’s gender	−.27 (0.60)	.65	0.76 (0.24-2.47)
Months since diagnosis	−.01 (0.02)	.74	0.99 (0.96-1.03)
Mini-Mental State Examination score	−.09 (0.06)	.17	0.92 (0.81-1.04)
Caregiver adoption status	.13 (1.01)	.90	1.1 (0.16-8.24)
Caregiver activated the app	1.6 (0.70)	.02	5.1 (1.29-19.99)
Constant	2.5 (2.83)	.29	12.4

**Table 4:** Baseline characteristics of participants who replied to the survey compared with participants who did not reply to the survey.

Characteristics		Participants who replied to survey (N=35)	Participants who did not reply to survey (N=77)	*P* value	
Age (years), mean (SD), range		68 (9.7), 52-86	69 (8.5), 39-83	.65	
**Gender, n (%)**					.84
	Women	15 (46)	33 (43)		
**Years of education, n (%)**					.92
	≤10	6 (18)	10 (13)		
	11-12	6 (18)	16 (21)		
	13-14	6 (18)	19 (25)		
	15-16	9 (27)	19 (25)		
	≥17	6 (18)	13 (17)		
**Diagnosis, n (%)**					.33
	Alzheimer disease	21 (64)	43 (56)		
	Vascular dementia	0	2 (3)		
	Dementia with Lewy bodies	1 (3)	0 (0)		
	Frontotemporal dementia	0	3 (4)		
	Mild cognitive impairment	4 (12)	5 (7)		
	Other^a^	6 (15)	21 (27)		
	Unresolved^b^	2 (6)	3 (4)		
Months since diagnosis, mean (SD), range		9 (12), 0-61	14 (15), 0-73	.06	
Mini-Mental State Examination score^c^, mean (SD), range		26 (4.1), 11-30	24 (4.4), 11-30	.12	

^a^For example, Huntington disease or stroke.

^b^Participants who were not diagnosed at the time of inclusion.

^c^Higher scores indicate higher attainment. The scores on the Mini-Mental State Examination are the most recent scores documented in participants’ medical records.

**Table 5:** Data from survey: participant’s and caregiver’s rating of the participant’s level of experience, skills, and need for help when using a tablet.

Participants’ and caregivers’ by-proxy rating^a^				Adopter^b^		Nonadopter^b^		*P* value
**Participant**								
	**Level of experience as tablet user, n (% within group)**						.49	
		Much experience	2 (15)		5 (28)			
		Some experience	7 (54)		5 (28)			
		Little experience	1 (8)		4 (22)			
		Novel user	3 (23)		4 (22)			
	**Skills as tablet user, n (% within group)**						.15	
		Uncomplicated	7 (54)		7 (44)			
		A little difficult	5 (38)		2 (12)			
		Quite difficult	1 (8)		5 (31)			
		Very difficult	0		2 (12)			
	**Help from others when using a tablet, n (% within group)**						.54	
		No help	5 (38)		6 (33)			
		A little help	6 (46)		5 (28)			
		Some help:	1 (8)		3 (17)			
		A lot of help	1 (8)		4 (22)			
**Caregiver by-proxy**								
	**Level of experience as a tablet user, n (% within group)**						.41	
		Much experience	3 (38)		4 (19)			
		Some experience	3 (38)		7 (33)			
		Little experience	1 (12)		7 (33)			
		Novel user	1 (12)		3 (14)			
	**Skills as a tablet user, n (% within group)**						.38	
		Uncomplicated	3 (37)		6 (30)			
		A little difficult	3 (37)		3 (15)			
		Quite difficult	2 (35)		6 (30)			
		Very difficult	0		5 (25)			
	**Help from others when using a tablet, n (% within group)**						.28	
		No help	2 (25)		3 (14)			
		A little help	4 (50)		8 (36)			
		Some help	2 (25)		4 (18)			
		A lot of help	0		7 (32)			

^a^Ratings cannot be compared between participants and caregivers because participant’s and caregiver’s rating does not refer to parallel cases.

^b^Answers were not complete to all questions; hence, the total number of answers on each question varied for both participants and caregivers.

In those cases where the app had not been activated by the participant or the person had stopped using it, the survey gave opportunity to comment on reasons for not using or adopting the app. These comments are summarized in themes (Textbox 1). The results indicated that both among participants and caregivers, common reasons were that the app did not fit their needs, some indicated the using the app was too early from the perspective of living with a progressive dementia disease, and others found it too difficult to use. Some indicated that they preferred using standard off-the-shelf software (eg, the calendar app that is preinstalled in the device), and others preferred to continue using a paper diary. Reasons for not using the app were, in some cases, also related to cognitive symptoms caused by the dementia disease (eg, some participants forgot to use the app or had lost the ability to use a tablet), indicating that this kind of AT was introduced too late for the person with a progressive disease.

**Participants’ and caregivers’ reasons for not using the app.**

Theme: participant intends to start using the app

- Participant quotes
  - I want to use the app, I just need to learn how to use it.
  - I will try to install the app.
  - I think I will start using it, the calendar would be nice to have.

Theme: using the app was found premature

- Participant quotes
  - It’s not relevant for me...yet.
- Caregiver quotes
  - It was too simple for him
  - I think we will wait until it is the right time to use it.

Theme: participant does not use the app because of dementia symptoms

- Participant quotes
  - I forget to use it.
  - I don’t think about using it...I forget.
  - Frankly, I have forgotten that I have ever heard of it.
- Caregiver quotes
  - He is not able to use the iPad anymore.
  - She has opened the app a few times, she but does not understand how to use it. It is too late to introduce it.
  - The problem is my wife forgets to use it.
  - Our dad probably got the app too late.
  - She feels under surveillance when using the app.

Theme: participant prefer to use other technology solutions

- Participant quotes
  - I am happy with the things I use already.
  - I use a computer for e-mails, calendar and things like that...I have an iPad, but I haven’t started using it yet.
  - When I read more about what the app could do I realised that I was covered by the things I already use, and I like using the things that I already know.
  - I prefer to use the calendar that is installed on my phone.
  - When I read more about what the app could do I realised that I was covered by the things I already use, and I like using the things that I already know.
  - I prefer to use the calendar that is installed on my phone.
- Caregiver quotes
  - He prefers to use the calendar which is installed on the iPad.

Theme: participant prefer to use nontechnology solutions

- Participant quotes
  - It is easier for me to use a paper diary.
  - I prefer my paper diary.
- Caregiver quotes
  - She finds it easier to use an ordinary paper diary.

### Use Patterns and Usability

To enable a more detailed analysis of use patterns of those participants who activated the app (N=65), they were split into 4 groups based on the number of days they had been users of the app (number of days from the first to last activity in the app, with a maximum of 90 days): short use (1-10 days), early abandonment (11-31 days), late abandonment (32-90 days), and adopter (≥90 days). These are summarized in Table 2.

**Figure 2:**
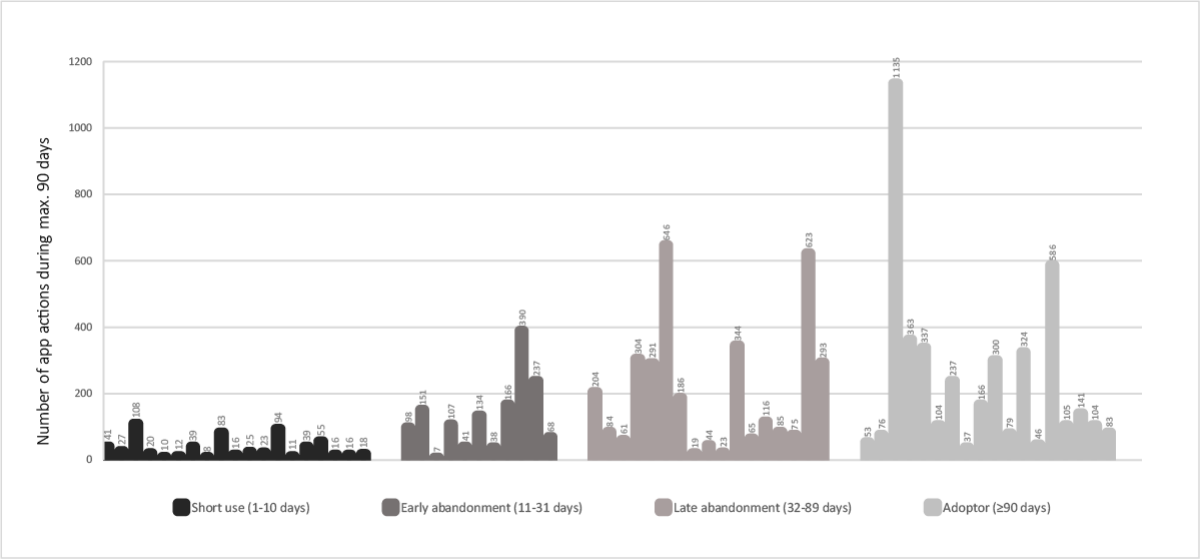
Number of activities in the app (maximum 90 days) for all participants who activated the app.

The number of activities in the app for the 4 groups of participants who had activated the app is summarized in Figure 2, which illustrates a clear tendency that a longer period of using the app generated more activities in the app; however, there was a large within-group variation.

**Figure 3:**
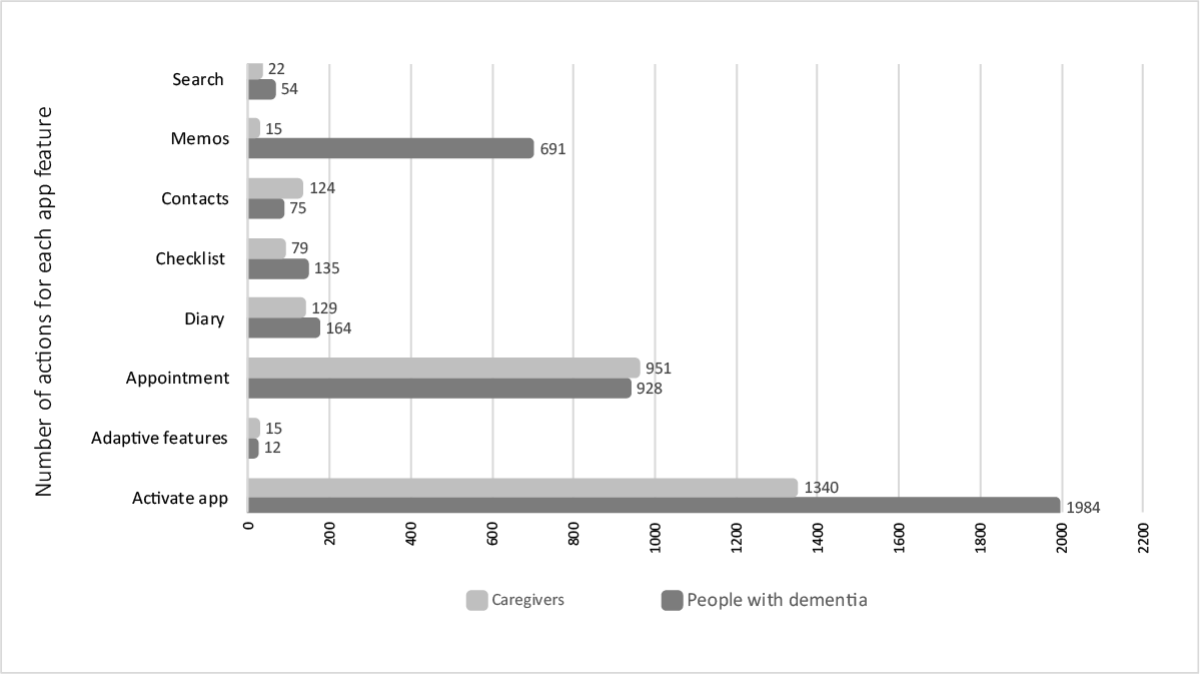
Log data from adopters illustrating what app functionalities were used.

To further assess if the content of the app was relevant to users or if adaptions were needed, data from adopters were further analyzed. The number of times each functionality was used by a participant or caregiver is illustrated in Figure 3.

The level of satisfaction with the app could be analyzed based on data from the USEdem questionnaire. Analysis of results (not illustrated) showed an overall average score of 40 (SD 9.4; range 21-55) for participants and 34 (SD 12; range 18-51) for caregivers, which indicated a generally positive rating of the app with regard to usefulness, satisfaction, and ease of use, but with large variation. For the participants, the score on the USEdem questionnaire was significantly higher for adopters (median 46) compared with nonadopters (median 38; U=5.5; *P*=.02; *r*=0.64). There was also a tendency toward higher scores on by-proxy replies from caregivers to participants who became adopters, but this difference did not reach statistical significance (*P*=.14).

## Discussion

### Principal Findings

The aims of this study were to investigate the applicability and usability of the ReACT app in a mixed group of people with dementia and to investigate user patterns, factors influencing adoption, and possible impact of caregiver involvement. The study also served as a process evaluation of both the app and methods for implementation.

The overall adoption rate was not high in this study, and whether it can be considered a successful adoption rate or not is hard to estimate since, to the best of the authors’ knowledge, no previous comparable studies have been conducted within the field of dementia research. The relatively low adoption rate is in line with the well-known challenge of nonadoption and nonadherence for digital health interventions across all medical fields, referred to as *the law of attrition* [13] as described in the Introduction section.

### Participant Profiles

According to the study objectives, a heterogeneous sample of people referred to a memory clinic were included in the study. As expected from a mixed population of people with dementia, most of the participants had a diagnosis of Alzheimer disease; however, the prevalence was higher among participants in this study compared with a general Danish population of people referred to a memory clinic [21], and participants were also, on average, younger compared with a general population of people referred to a memory clinic [21].

The relatively young age of participants could reflect an age or generation-related higher frequency of using tablets and apps among middle-aged and younger seniors compared with older generations [22]. However, it is also important to notice the relatively wide age range among participants, and there was no significant age difference between adopters and nonadopters, underlining the importance of not letting age be a determinant when presenting AT to people with dementia.

### Disease-Related Factors Had Influence on Adoption

The MMSE scores, indicating the level of cognitive function, were generally higher among participants than in a general Danish population of people referred to a memory clinic [21]. These results indicate that participants who were interested in using the app, and hence included in the study, were generally at an early stage of dementia, and this was consistent with the target group of the app. There was no significant difference in MMSE scores for adopters and nonadopters, indicating that results from the cognitive screening tests should not guide introduction of AT to people with dementia. However, time since diagnosis was statistically significant and can be considered a pseudo marker for disease severity. This finding shows that timely introduction of AT can be of great importance for successful adoption. Comments from the survey indicated that reasons for nonadoption in some cases were that it was considered too early to use AT tailor-made for people with dementia and standard off-the-shelf software met the participant’s current needs. This preference to use off-the-shelf technology among people with dementia and family caregivers has also been observed in other studies [23,24]. In other cases, comments revealed that the app was introduced too late at a stage where the participant was no longer able to learn how to use it or had lost the ability to use a touchscreen device. This notion of timely introduction of technology is in line with the general emphasis on the importance of timely delivery of support and interventions for people with dementia [25,26], and it underlines the importance of providing clear and user-centered labeling of AT for people with dementia, enabling users and caregivers to choose a solution that fits their current needs and resources and professionals to give individualized advice on the use of AT, and thereby provide genuinely person-centered AT to people with dementia.

As illustrated in Figure 1, the app was designed to be adaptable and could be tailored to fit individual preferences and skills. The idea was the app could either from the start be adapted to a person with a more advanced stage of dementia or over time gradually be adapted to fit the changing needs because of progressive cognitive symptoms. As summarized in Figure 3, analysis of log data from adopters showed that adaptive features of the app were in total only activated 12 times by participants and 15 times by caregivers, indicating that, perhaps, there is a need to promote these features more strongly. Future studies will be needed to explore if these adaptable features are suitable to fit the changing needs of a user or if such features should be further adapted.

In general, research is needed to explore how long-term adherence to technology can be supported among people with dementia and to define realistic goals for long-term adherence among this group. It is also important to consider whether progressive cognitive symptoms might imply that aiming for long-term adherence is too ambitious. Various models and frameworks have been proposed for successful innovation, design, and implementation of AT [27] and electronic health (eHealth) solutions [28,29]. The holistic framework to improve the uptake and impact of eHealth technologies proposed by van Gemert-Pijnen et al [29] has been applied within the field of dementia [30]. In future studies, it is essential to explore how such frameworks can be further integrated into the innovation and implementation of lifecycles of AT for people with dementia. It is, for instance, important to consider the progressive nature of dementia diseases and how it affects AT needs and skill over time and to include such variable disease-related factors in these frameworks.

### Caregiver Involvement

Our results clearly indicated that caregiver involvement in using the app could influence participants’ adoption of it. These results are in line with other studies finding caregiver engagement important for the everyday use of technology among people with dementia [23]. The benefits of caregiver involvement have implications for the design and implementation of AT for people with dementia. AT should be designed to promote convenient and flexible caregiver support in a way that is feasible and acceptable for both the person with dementia and the caregiver. There are, however, important issues of privacy and ethics that need to be addressed when designing and implementing AT [31]. The ReACT app was designed as a Web-based app, it could be accessed from several devices simultaneously, and this allowed flexible caregiver support. Our design also allows the person with dementia to decline caregiver’s support because caregiver’s access could be deactivated; this option was never used during the study. In many cases, the caregiver undoubtedly also supported the use of the app alongside the participant without accessing their parallel login, but our data do not provide enough insight into this use pattern.

The benefits of caregiver involvement also highlight the need to provide information and guidance to caregivers on how they can best support people with dementia in using AT. In this study, this was done by providing written and Web-based material giving advice on caregiver involvement. The need to consider methods to support caregiver involvement has also been discussed by others [23], stressing the need to address both the person with dementia and caregivers and their mutual cooperation on AT use when designing methods for the implementation of AT.

It is important to acknowledge, however, that in some cases, participants adopted the app and were high-frequency users with minimal or no caregiver involvement, again indicating a large variation among adopters and stressing that the person with dementia should be addressed as the main user of AT for people with dementia.

The regression analysis showed that none of the participants’ background characteristics could predict participants’ adoption status, and overall, the regression to predict adoption was relatively low in predictive power (Nagelkerke *R^2^*=0.173) underlining that a complex set of personal and contextual features influence adoption of AT, making it difficult to predict adoption and adherence. It also underlines the limited applicability of models trying to predict the use of AT among this group of users, which have been proposed by others [32].

### Usability and Use Patterns

Data from the surveys revealed that there were no significant differences between adopters and nonadopters when it came to how much experience they had using a tablet, their skills when using it, and how much help they needed to use it. Some of the adopters were even characterized as novel users, indicating that the app is applicable for a varied group of users and can be used despite having a low level of tablet skills, which has also been demonstrated in a previous pilot study [17]. This finding is similar to other studies demonstrating the accessibly and user-friendliness of tablet computers and app-based interventions for people with dementia [33,34].

Use patterns among all participants who activated the app are illustrated in Figure 2, showing considerable variation in how intensively the app is used in all groups. Interestingly, among nonadopters who abandoned the app late (after 32-89 days of use), there were users who used the app quite intensively. The reasons for their late abandonment of the app were not revealed by our data because most were lost to follow-up in the survey. These log data indicate that it can be of great importance that both people with dementia and caregivers are provided support not just at the beginning of a self-applied intervention similar to this but also during the intervention to support the continued use of the app. Further studies are needed to address what kind of support is needed and how it is best delivered.

The results from the USEdem questionnaire revealed a relatively high satisfaction with the app among participants and caregivers, but with some variation, and participants who became adopters rated it significantly higher than nonadopters. Results from caregivers were generally less positive, and this should, of course, be investigated further. However, data quality on this questionnaire was limited because of the small number of replies.

Log data showed that all functionalities in the app were used, as illustrated in Figure 3. There was, of course, variation, reflecting that some functionalities, such as appointment and memos, were by nature more frequently used compared with others (eg, search). Consequently, this part of the process evaluation did not imply major changes of the app.

### Limitations

This study has several limitations that should be acknowledged. Participants were recruited from memory clinics, and consequently, only people who have sought an examination and had contact with a memory clinic were included. This limitation will be addressed in a subsequent study with open access to the app. In addition, staff could have been biasing inclusion. Although information on the study was generally available in the clinics, inclusion was also promoted by staff, and they could have been directing information to subgroups of participants, based on common ideas of who can benefit from using AT (eg, younger participants or those with mild cognitive symptoms).

In addition, a number of participants did not have a dementia diagnosis; hence, describing participants as a group of people with dementia could be considered imprecise. There was also a quite large proportion of participants who were categorized as *other*, some had a nonspecific dementia diagnosis or stroke, and many of these did not become adopters. This could indicate a risk of bias from participants being included whose needs did not match the design and functionalities of the app. The study did, however, aim to apply the intervention to a broad group of potential end users, allowing their own need and motivation to guide inclusion, rather than specific disease-related factors. The relatively large proportion of *others* who were nonadopters underline the need for clear and user-centered labeling of AT for people with dementia as discussed previously.

Another limitation was that the app could only be used on a series of tablets (iPads) during this study, and this, of course, excluded potential participants who had access to other kinds of tablets. For practical and financial reasons, a specific type of tablet had to be selected when designing the app for this study, and the selection was based on various factors (eg, the iPad was the most common tablet in Denmark) [35].

Data included in this study were generally rich and provided interesting results and observations, but there were also limitations in relation to data quality and quantity. Disease-related information was only obtained from medical records, and no pre-post measures were applied. This could be changed in future studies. However, as shown by previous studies [36,37], it is difficult to capture the essence of such interventions by applying traditional outcome measures addressing, for instance, cognition, daily activities, or quality of life. In line with the broader field of psychosocial interventions, there is a great need to develop outcome measures that are more appropriate for the specific intervention [38]. In addition, applying a survey to this group of end users causes limitations. Completing a survey can be challenging for a person with dementia and impossible for those more severely affected by their disease. Our results showed that months since diagnosis was close to significance (*P*=.06) when comparing participants who replied to the survey with those who did not reply, indicating a risk that data could be biased by being collected from participants who were less severely affected by their disease. In addition, the amount of survey data obtained from caregivers was limited. Although data quantity was low, the survey did contribute valuable data. In future studies, we suggest that feasibility of surveys must be considered, but not generally abandoned as a method for data collection among this group of participants.

The study was designed to provide separate log data from participants and caregivers; however, this does not reveal all details on how the app is used in real life, for example, caregiver support could be more intense than revealed by current data. In future studies, richer data can be obtained by including more qualitative data (eg, by interviewing participants and caregivers). The aim of the study was, however, to obtain detailed data from a large group of potential users of the app, and this could be obtained by the mixed method design, which is also used to evaluate the use of apps in other fields of health care [39]. Analysis of extensive log data from participants and caregivers brings an important new methodology to this field of research.

### Conclusions

The results from this study were in line with the general well-known challenges of nonadoption and nonadherence to digital health interventions. However, for those who adopted the app, results showed that the ReACT app was applicable and useful for a mixed population of people with dementia and that the methods used for deployment and self-applied implementation were applicable for this group of end users. The study clearly demonstrated the benefits of applying mixed methods when accessing applicability, usability, and effectiveness of AT. The analysis of detailed log data contributed valuable insights into use patterns and allowed a detailed analysis of factors influencing adoption. In addition, it provided detailed data used in the process evaluation and validation of the ReACT app. To the best of the authors’ knowledge, this study is the first to include such a large and rich dataset from the everyday use of AT among people with dementia and their family caregivers.

The results from the study revealed factors that could influence the adoption of AT among people with dementia. Timely introduction of AT and support from caregivers had significant influence on whether participants adopted the ReACT app. However, data also revealed great variation among adopters when it came to personal, disease-related, and contextual factors, and the predictive value of caregiver involvement was small. This underlines that adoption of AT among people with dementia is influenced by a complex set of personal and contextual factors, which is made even more complex by the changing needs imposed by living with a progressive dementia disease. This complexity and variability restrain the extent to which adoption and adherence to AT can be predicted and emphasize the importance of incorporating this wide range of changeable factors when designing and implementing AT for people with dementia.

## Abbreviations

AT: assistive technology
eHealth: electronic health
ICT: information and communication technology
MMSE: Mini-Mental State Examination
ReACT: Rehabilitation in Alzheimer's disease using Cognitive support Technology
